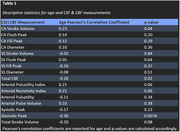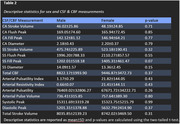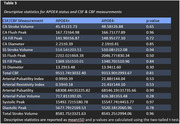# Differences in cerebrospinal fluid and cerebral blood flow measurements by age, sex, and APOE4 status in older adults

**DOI:** 10.1002/alz.091136

**Published:** 2025-01-09

**Authors:** Yilei Dong, Ashwin Sakhare, Joy Stradford, Teresa Monreal, A. Lisette Isenberg, Judy Pa

**Affiliations:** ^1^ Alzheimer's Disease Cooperative Study (ADCS), University of California, San Diego, La Jolla, CA USA; ^2^ Laboratory of Neuro Imaging, Stevens Neuroimaging and Informatics Institute, Keck School of Medicine, University of Southern California, Los Angeles, CA USA; ^3^ Neurosciences Graduate Program, University of California, San Diego, La Jolla, CA USA

## Abstract

**Background:**

The glymphatic system is important for clearing waste and transporting nutrients throughout the brain, but it’s still unknown how cerebrospinal fluid (CSF) and cerebral blood flow (CBF) contribute to Alzheimer’s Disease (AD). Phase‐contrast MRI (PC‐MRI) has been shown to reliably measure CSF and CBF at the cerebral aqueduct (CA) and second and third cervical vertebrae (C2‐C3) non‐invasively. The purpose of this study is to demonstrate CSF and CBF flow dynamics variations by age, sex, and APOE4 status in older adults.

**Method:**

Seventy‐nine healthy older adults (66.9±7.1 years old, 47 women) were recruited for this study from two larger clinical trials. All subjects received an MRI scan at baseline. CSF and CBF measurements were obtained using a 2D cine PC‐MRI pulse sequencing with retrospective cardiac gating. Measurements for CSF and CBF flow were taken at the CA, subarachnoid space (SS), and arteries between the C2‐C3. Correlation coefficients were calculated for age and CSF and CBF flow measurements, while mean and standard deviations of CSF and CBF flow measurements were calculated for sex and APOE4 status.

**Result:**

Age: For the CA, stroke volume (r=.23, p=.04) and total CBF (r=‐.26, p=.02) were significantly associated with age. For arteries, there was a non‐significant trend‐level association between age and arterial pulsatility index (r=.21, p=.06), arterial resistive index (r=.21, p=.06), and total stroke volume (r=‐.20, p=.08).

Sex: Though non‐significant, there was a trend towards significance for women to have a greater SS fill peak than men (p=.07). In general, women had higher absolute values for CSF and CBF measurements, but they were largely not significant.

APOE4 Status: There was no significant difference between APOE4 carriers and non‐carriers for any CSF and/or CBF measurements.

**Conclusion:**

This study shows that across age, sex, and APOE4 status, there are variations in CSF and CBF measurements for participants that may contribute to glymphatic clearance and AD.